# Genomic and mutational analysis of *Pseudomonas syringae* pv. *tagetis* EB037 pathogenicity on sunflower

**DOI:** 10.1186/s12866-024-03685-8

**Published:** 2025-01-24

**Authors:** Jude E. Maul, John Lydon, Dilip Lakshman, Colin Willard, Hyesuk Kong, Daniel P. Roberts

**Affiliations:** 1 USDA-ARS, Sustainable Agricultural Systems Laboratory, Beltsville Agricultural Research Center, Beltsville, MD 20705 USA; 2 USDA-ARS, Office of National Programs, George Washington Carver Center, Beltsville, MD 20705 USA; 3 Present Address: Food and Drug Administration, Center for Biologics Evaluation and Research, Silver Spring, Beltsville, MD 20993 USA

**Keywords:** Apical chlorosis, Disease, Effectors, *Pseudomonas syringae* pv. *tagetis*, Sunflower, Type III secretion system, Type IV secretion system

## Abstract

**Background:**

*Pseudomonas syringae* pv. *tagetis* (*Pstag*) causes apical chlorosis on sunflower and various other plants of the Asteraceae family. Whole genome sequencing of *Pstag* strain EB037 and transposon-mutant derivatives, no longer capable of causing apical chlorosis, was conducted to improve understanding of the molecular basis of disease caused by this pathogen.

**Results:**

A tripartite pathogenicity island (TPI) for a Type III secretion system (T3SS) with the complete *hrp-hrc* gene cluster and conserved effector locus was detected in the *Pstag* genome. The exchange effector region of the TPI contained genes potentially functioning in detoxification of the environment as well as two integrases, but no previously described T3SS effector homologues. In all, the *Pstag* EB037 genome contained homologues for at least 44 T3SS effectors with 30 having known functions. Plasmids similar with pTagA and pTagB of *P. syringae* pv. *tagetis* ICMP 4091 were also identified in the *Pstag* genome. The pTagA-like plasmid contained a complete Type IV secretion system (T4SS) with associated putative killer protein. Mutational analysis using transposon insertions within genes functioning in the T3SS and T4SS confirmed the role of both secretion systems and these plasmids in apical chlorosis. Transposon mutagenesis identified an additional 22 genes in loci, including two more plasmid-bound loci, involved in apical chlorosis on sunflower; some with known importance in other plant or animal pathosystems.

**Conclusions:**

Apical chlorosis disease caused by *Pstag* EB037 is the result of a complex set of mechanisms. This study identified a TPI and homologues for at least 44 T3SS effectors, 30 of which with known functions in disease, and another 20 genes in loci correlated with apical chlorosis on sunflower. Two plasmids were detected that were correlated with apical chlorosis disease, one of which contained a complete T4SS that was correlated with disease. To our knowledge, we provide the first direct evidence for a T4SS functioning in disease by a pathogenic *P. syringae* strain.

**Supplementary Information:**

The online version contains supplementary material available at 10.1186/s12866-024-03685-8.

## Background

*Pseudomonas syringe* consists of approximately 60 pathovars that collectively cause disease on numerous monocot and dicot crops [14]. These bacteria persist as epiphytes colonizing leaves and other exterior plant parts and as endophytes where they colonize the intercellular plant apoplast and cause disease [83, 127]. Survival on exterior, aerial plant surfaces requires the ability to withstand biotic and abiotic stress (antimicrobial compounds, oxidative stress, osmotic stress, UV light) and acquire nutrients [65, 120]. Colonization of the intercellular apoplast requires entering the leaf, overcoming PAMP (pathogen associated molecular pattern)—triggered immunity (PTI) and effector-triggered immunity (ETI), and acquisition of nutrients [64, 65, 78, 79, 127]. Production of both flagellar and Type IV pili for motility, the Type III secretion system (T3SS) and T3SS effectors to overcome plant immunity, viscous extracellular polysaccharide for biofilm formation for protection against challenging environments, and catalases and other enzymes for detoxification of harmful compounds were important for disease caused by the *P. syringae* pathovars that have been studied in depth [26, 40, 52, 89, 127].

*Pseudomonas syringae* pv. *tagetis* (*Pstag*) infects various plants of the Asteraceae family, including sunflower [60], resulting in apical chlorosis in developing leaves and stunting of the plant [69]. *Pstag* occupies canonical *P. syringae* Phylogroup 6 based on analysis of a limited number of strains including the pathotype ICMP 4091 [9, 127]. Isolates from canonical phylogroups typically contain tripartite pathogenicity islands (TPI) with the *hrp—hrc* gene cluster (encoding a T3SS) flanked by the conserved effector locus (CEL) and the exchangeable effector locus (EEL) [1, 127]. Isolates belonging to canonical phylogroups also typically have additional virulence factors including ice nucleation, auxin production, and production of exopolysaccharide (EPS) alginate [8, 16, 44, 65, 107]. Apical chlorosis is due, at least in part, to the activity of tagetitoxin, a phytotoxin specific to this pathovar [99]. The bicyclic molecule tagetitoxin targets the plant apex where it inhibits chloroplast RNA polymerase resulting in plastids that have no, or poorly developed, thylakoid membranes [3, 45, 73, 76, 81, 82, 132]. There is some evidence that *Pstag* requires *exbD* and a functional TonB system for tagetitoxin export [59]. Little more can be posited regarding the molecular basis of disease caused by isolates of *Pstag* other than the above general information gleaned from studies with other pathovars.

Here we present the sequence of the *Pstag* EB037 genome. We also sequence *Pstag* EB037 derivative mutants impacted in the ability to cause apical chlorosis and perform comparative functional analysis of the EB037 genome to deepen our understanding of the genes and traits required for *Pstag* to cause disease.

## Results

### General features of the *Pstag* EB037 genome

The sequenced *Pstag* EB037 genome consisted of 10 scaffolds/contigs containing 6,274,278 bases with a 58.2% GC content (Table 1). There were 5580 total predicted genes of which 5512 were protein-coding genes and 68 of which were non-protein-coding genes. Among the predicted genes 3540 were assigned a function and 37 were pseudogenes. There were eight predicted rRNA genes and 63 predicted tRNA genes. Strain EB037 genome size, percent GC content, total number of genes, protein coding genes, and tRNA genes were similar with *Pstag* ICMP 4091, *P. syringae* pv. *syringae* B728a, *P. savastanoi* pv. *phaseolicola* 1448A, and *P. syringae* pv. *syringae* DC3000 (Table 1). Strain EB037 was like strains ICMP 4091, B728a and DC300 regarding the number of pseudogenes. Both *Pstag* strains had substantially less rRNA genes than the other three strains. Strain EB037 also contained two plasmids that were similar with pTagA and pTagB from *Pstag* ICMP 4091. In strain EB037 the pTagA- and pTagB-like plasmids were 47,148 and 73,120 bp in size, respectively (Supplementary Tables S1 and S2), while in strain ICMP 4091 pTagA and pTagB were 56,569 bp and 46,781 bp in size, respectively.

**Table 1:** Genome characteristics of *Pseudomonas syringae* pv*. tagetis* EB037 and similar strains^a^

Genome characteristics^b^	*P. syringae* pv. *tagetis* EB037	*P. syringae* pv. *tagetis* ICMP 4091	*P. syringae* pv. s*yringae* B728a	*P. savastanoi* pv. *phaseolicola* 1448A	*P. syringae* pv. *tomato* DC3000
Size (bp)	6,274,278	6,042,642	6,093,698	6,112,448	6,538,260
Percent GC content	58.2	58.2	59.2	57.9	58.3
Number of scaffolds	10	131	1	3	3
Total number of genes	5580	5522	5173	5766	5842
Protein-coding genes	5512	5412	5089	5165	5619
Non-coding genes	68	110	84	84	223
rRNA genes	8	4	16	16	15
tRNA	63	51	64	64	63
Pseudogenes	37	42	47	601	29

^a^Data is from this study for *P. syringae* pv. *tagetis* EB037, Thakur et al. [116] for *P. syringae* pv. *tagetis* ICMP 4091, Fei et al. [35] for *P. syringae* pv. *syringae* B728a, Joardar et al. [55] for *P. savastanoi* pv. *phaseolicola* 1448A, and Buell et al. [13] for *P. syringae* pv. *tomato* DC3000

^b^Data for comparison genomes retrieved from www.ncbi.nlm.nih.gov/assembly/ on May 20, 2019

Dot plot analyses were performed comparing homology and gene order of strain EB037 with that of strains ICMP 4091, B728a, and 1448A and with that of *Pseudomonas putida* KT2440 as a negative control (Supplementary Fig. S1). Dot plot analysis indicated that the genomes of *Pstag* strains EB037 and ICMP 4091 had substantial homology but were highly dissimilar in terms of gene arrangement and order. This may be due to the high number of contigs (131) associated with the genome submission of ICMP 4091. There was substantial homology and synteny between strains EB037 and B728A with some sequence rearrangements. Genomes of strains EB037 and 1448A also exhibited substantial homology, but there were numerous sequence rearrangements and the annotation between strains was reversed. The comparison of genomes of *Pstag* EB037 and the control, *P. putida* KT2440, revealed very little homology or synteny.

### *Pstag* EB037 disease-related genes identified through genome mining

**Fig. 1:**
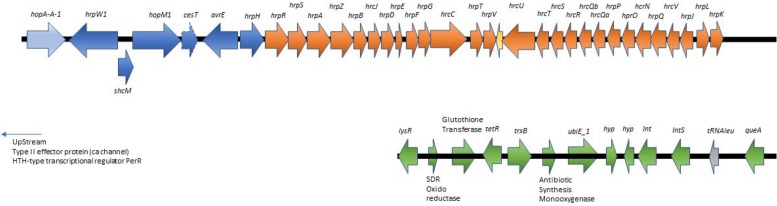
*Pseudomonas syringae* pv. *tagetis* EB037 tirpartite pathogenicity island. Arrows indicate direction of transcription of individual genes. *hopAA-1*, Type III secretion system (T3SS) effector protein HopAA-1; *hrpW1*, harpin HrpW1; *hopM1*, T3SS effector protein HopM1; *cesT*, chaperone protein CesT; *avrE*, Type III secretion transcription activator, regulates HrpL; *hrpS*, NtrC-like transcription activator, regulates HrpL; *hrpA*, needle protein HrpA; *hrpZ*, helper protein HrpZ; *hrpB*, inner rod protein HrpB; *hrcJ*, inner membrane ring protein HrcJ; *hrpE*, ATPase complex protein HrpE; *hrpD*, ATPase complex factor HrpD; *hrpF*, negative regulator protein HrpF, stabilizes HrpA; *hrpG*, regulatory protein, suppresses negative regulation by HrpV; *hrcC*, component of Type III secretion machinery; *hrpT*, secretin pilotin protein HrpT; *hrpV*, HrpV protein, negatively regulates HrpR and HrpS; *hrcU*, autoprotease HrcU; *hrcT*, inner membrane component protein HrcT; *hrcS*, inner membrane component protein HrcS; *hrcR*, inner membrane component protein HrcR; *hrcQb*, cytoplasmic ring protein HrcQb; *hrcQa*, cytoplasmic ring protein HrcQa; *hrpP*, needle-length regulator protein HrpP; *hrpO*, ATPase complex protein HrpO; *hrcN*, ATPase complex protein HrcN; *hrpQ*, inner membrane ring protein HrpQ; *hrcV*, export gate protein HrcV; *hrpJ*, switch regulator protein HrpJ; *hrpL*, sigma factor, master regulator of majority of *hrp, hrc, hop,*and *avr* genes; *hrpK*, translocation pore protein HrpK; *lysR*, LysR-type transcriptional regulator; SDR oxidoreductase; glutathione transferase; *tetR*, TetR family regulatory protein; *trxB*, thioredoxin reductase; *ubiE1*, ubiquinone/menaquinone biosynthesis C-methyltransferase; *hyp*, hypothetical protein; Int, integrase; IntS, integrase; *tRNA*^*leu*^, encodes tRNA.^Leu^; *queA,* S-adenosylmethionine:tRNA ribosyltransferase-isomerase [19, 25, 122, 126]

**Fig. 2:**
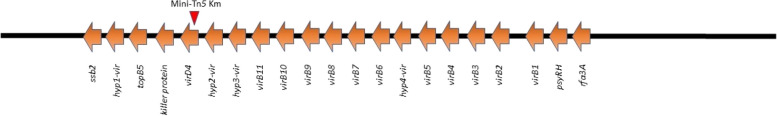
*Pseudomonas syringae* pv. *tagetis* EB037 Type IV secretion system locus. Arrows indicate direction of transcription of individual genes. *rfa3A,* replication factor A protein; *virB1,* peptidoglycan hydrolase; *virB2,* pilin protein; *virB3,* inner membrane complex protein; *virB4,* inner membrane complex ATPase; *virB5*, inner membrane complex pilus-tip protein; *hyp4-vir,* hypothetical virulence protein; *virB6,* polytopic inner membrane core protein; *virB7,* outer membrane complex lipoprotein; *virB8,* inner membrane complex bitopic transmembrane protein; *virB9,* outermembrane complex protein; *virB10,* inner membrane complex/outer membrane complex bitopic transmembrane protein; *virB11,* cytoplasmic/inner membrane complex ATPase; *hyp3-vir,* hypothetical virulence protein; *hyp2-vir,* hypothetical virulence protein; *virD4,* Type IV coupling protein, ATPase; killer protein, predicted function in ubiquination and cell apoptosis; *topB5,* DNA topoisomerase B protein; *hyp1-vir,* hypothetical virulence protein; *ssb2,* replication factor A protein 2 [108]

Numerous homologues of disease-related genes were identified via analysis of the EB037 genome sequence (Table 2; Figs. 1 and 2). These included a canonical TPI containing a *hrp-hrc* gene cluster, with genes for the complete T3SS injectosome, flanked on one side by the CEL and on the other by an EEL. The CEL contained homologues for the highly conserved effector genes *hopAA1-1*, *hopM1*, and *avrE* [1]. The EEL was bracketed by *hrpK* and *tRNA*^*leu*^ and contained genes for an SDR oxidoreductase, glutathione transferase, an antibiotic synthesis monooxygenase, and two hypothetical proteins as well as *trxB*, *ubiE1, lysR,* and *tetR* (Fig. 1). Forty-one homologues of T3SS effector genes (Table 2) were detected in the genome of *Pstag* EB037 outside of the TPI.

**Table 2:** Disease-related genes identified in the *Pseudomonas syringae* pv. *tagetis* EB037 genome

**Gene**^**a**^	**Description**^**b**^	**Reference**
	**T3SS effectors**	
*avrB3*	AvrB3; jasmonate signaling manipulation	[127]
*avrE*	AvrE down-regulated expression of plant NHL13 gene	[129]
*avrPto1*	AvrPto1 targeted FLS2, EFR; suppressed PTI	
*avrRpm1*	AvrRpm1 targeted RIN4; interfered with RIN4 complex	[127]
*hopA1*	HopA1 targeted EDS1; interfered with EDS1 and proteasome	[127]
*hopAA-1*	HopAA-1; host target unknown	[85]
*hopAB2*	HopAB2 (or AvrPtoB); targeted ABA, RIN4, R_HopAD1_; interfered with PTI, ETI	[127]
*hopAD1*	HopAD1 elicited immunity-associated cell death	[122]
*hopAF1*	HopF1 targeted MTN1 and MTN2; blocked ethylene induction	[127]
*hopAG1*	HopAG1; has putative Nudix domain	[20, 33]
*hopAH1*	HopAH1; expression in planta uncertain	
*hopAH2-1*	HOPAH2-1; glycosyl hydrolase 5 (cellulase) family	(Uniprot ID: S6QBC6_PSESF)
*hopAH2-2*	HopAH2-2; (2 genes) expression in planta uncertain	[15]
*hopAM1-2*	HopAM1-2; (or AvrPpiB1); Enhanced virulence during drought	[127]
*hopAO1*	HopAO1 interfered with proteasome	[127]
*hopAP1*	HopAP1 (or HopPtoH2); secretion not demonstrated	[98]
*hopAQ1*	HopAQ1; suppression class II effector	[46]
*hopAR1*	HopAR1 (or AvrPphB); targeted BIK1, PBS1, PBLs; interfered with PTI	[15, 127]
*hopAS1*	HopAS1 contributed to virulence in tomato	[110]
*hopAZ1*	HopAZ1 suppressed callose deposition	[74]
*hopB1*	HopB1 cleaved BAK1; interfered with PTI	[62, 127]
*hopC1*	HopC1 contributed to virulence of DC3000	[123]
*hopD*	HopD targeted transcription factor NTL9; suppressed ETI	[10]
*hopE1*	HopE1 targeted MAP65; microtubule process	[127]
*hopG1*	HopG1 targeted kinesin; interfered with actin and proteasome processes	[127]
*hopH1*	HopH1 contributed to virulence	[123]
*hopJ*	HopJ interacted with GTP cyclohydrolase 1	(STRING-DB*)
*hopK1*	HopK1 targeted chloroplast processes	[127]
*hopM1*	HopM1 down-regulated expression of plant NHL13 gene; impacted water balance	[127, 129]
*hopN1*	HopN1 targeted chloroplast PsbQ	[127]
*hopO1-1*	HopO1-1 increased proteasomal degradation of PD-linked proteins	[7, 97]
*hopO1-2*	HopO1-2 contained chloroplast transit peptide	[68]
*hopO1-3*	HopO1-3 expression in planta uncertain	[15]
*hopQ1*	HopQ1 interfered with cytokinin processes	[127]
*hopR1*	HopR1 masked HopQ1 recognition	[15]
*hopS1*	HopS1 subfamily alleles are now HopO	[29]
*hopS2*	HopS2 suppressed HopA1-dependent HR in presence of Type III chaperone	[46]
*hopV1*	HopV1; avirulence determinant in *Nicotiana benthamiana*	[123]
*hopT1-2*	HopT1-2; expression during host infection uncertain	[15]
*hopT2*	HopT2; expression in planta uncertain	[15]
*hopU1*	HopU1 targeted GRP7 and GRP8 mRNA-binding proteins; suppressed immunity	[127]
*hopY1*	HopY1 (also HopPtoY); HopY1 targeted RIN4; suppressed PTI	[23]
*hopZ3*	HopZ3 manipulated jasmonate signaling	[127]
*xopAD*		
	**T3SS-associated proteins**	
*schA*	SchA*;* HopA1 chaperone	[54]
*schM*	SchM; HopM1 chaperone	[66]
*schF*	SchF; T3SS chaperone	
schO1	SchO1; T3SS chaperone	[66]
*schS1*	SchS1; T3SS chaperone	[66]
schV	SchV; T3SS chaperone	
*cesT*	CesT; T3SS chaperone	[86]
*hopAB2*	HopAB2 (or AvrPtoB); has E3 ubiquitin-ligase domain	[105]
hrpA2	T3SS helper HrpA2	
*hrpW1*	Harpin HrpW1	[61]
*hopAJ1*	T3SS helper HopAJ1	[92]
hrpK1	T3SS helper HrpK1	[61]
*hrpA1*	T3SS helper HrpA1	[61]
	**Potential function in degradation of plant cell-wall**	
*copABCD*	Multi-copper polyphenol oxidase; functions in resistance to copper ions	[51]
*HcaA*	Hydroxycinnamoyl-CoA hydratase-lyase; resistance to hydroxycinnamate substrates	[93]
*XanB2*	Chorismate-pyruvate lyase; involved in antioxidant activity	[136]
*pel*	Pectate lyase; degrades polygalacturonic acid in plant cell wall	[50]
*pehA*	Polygalacturonase ; degrades polygalacturonic acid in plant cell wall	[101]
*pem*	Pectin esterase; functions in degradation of pectin in plant cell wall	[11]
	Polysaccharide lyase family 7 protein (2 genes); function in pathogenesis	[71]
*xynA*	Endo-1,4-ß-xylanase; influences virulence	[104]
*aprA*	Zn-dependent protease; involved in virulence	[36]
	**Other**	
*bvgS*	Virulence sensor BvgS in *Bordetella* sp.	
*bvgA*	Positive transcription regulator BvgA	[84]
*exbD*	TonB (disease associated)	[59]
*gacA*	Response regulator of *gacA/S* TCS (plant disease associated)	[18]
*gacS*	Sensor histidine kinase of *gacA/S* TCS (plant disease associated)	[18]
killer protein gene	T4SS locus; predicted function in ubiquination, cell apoptosis	
*phoP*	TCS protein PhoP (virulence associated, *Pseudomonas aeruginosa*)	[43]
*phoQ*	Sensor histidine kinase PhoQ (virulence associated, *P. aeruginosa*)	[43]
*rcsC*	Sensor histidine kinase RcsC. Regulates flagellum synthesis (virulence associated)	[36, 39]
*sugA*	Trehalose transport permease SugA (virulence associated *Mycobacterium tuberculosis*)	[38]

*ABA* abscisic acid, *BAK1* BRASSINOSTEROID INSENSITIVE 1-associated receptor kinase1, *BIK1 Botrytis*-induced kinase 1, *EDS1* protein contributing to both PTI and ETI, *EFR* elongation factor Tu receptor, *ETI* effector-triggered immunity, *FLS2* flagellin-sensitive 2, *GRP* glycine-rich RNA-binding protein, *MAP65* microtubule-associated protein 65, *MTN* methylthioadenosine nucleosidase proteins required for PAMP-induced ethylene production, *PBL27* PBS1-like 27, *PBS1* AvrPphB susceptible 1, *PD* plasmodesmata, *PTI* pattern-triggered immunity, *PsbQ* photosystem II subunit Q, plant PsbQ mutants display diminished ROS production, *R*_*HopAD1*_ R protein recognizing HopAD1, *RIN4* RPM1-interacting protein 4, *T3SS* Type III secretion system, *T4SS* Type IV secretion system, *TCS*, two-component system

^a^Genes/proteins comprising the T3SS injectosome are described in Fig. 1. Genes/proteins comprising, and associated with, the T4SS locus are described in Fig. 2

^b^Plant proteins/hormone/process interacted with or modified by effector

The pTagA-like plasmid contained all 12 genes (*virB1* through *virB11*, *virD4*) of a canonical Class A Type IV secretion system (T4SS) (Fig. 2; Supplementary Table S1) encoding the core complex, the inner membrane complex, ATPases of the inner membrane complex, and the pilus [108]. *virD4* was detected downstream of the *virB* gene cluster. Hypothetical *vir* genes were detected within, or nearby, the *virB* cluster. A gene homologue for a killer protein was associated with the *virB* gene cluster as well as genes potentially functioning in conjugation (*virD4, topB5, rfa3A*) [27, 67]. A site-specific recombinase gene (*xerC*) was also identified on the pTagA-like plasmid. The pTagB-like plasmid contained components of a T4SS as well as several site-specific recombinase genes (*xerC*, *xerD*) and a gene for cysteine synthase (Supplementary Table S2).

Genes potentially functioning in degradation of the plant cell wall were detected (Table 2). Additional homologues of disease-related genes from *P. syringae* and other plant and animal pathosystems are listed in Table 2 including regulatory genes *bvgA*, *bvgS*, *phoP*, *phoQ*, and *rcsC* [2, 36, 39, 43, 84]. Genes encoding the ice nucleating protein (*ina*) and indoleacetic acid (IAA)-lysine synthetase (*iaaL*) were not detected in the *Pstag* EB037 genome unlike other Phylogroup 6 strains [127].

### *Pstag* EB037 disease-related loci identified through mutational analysis

Tox-17 and Tox-18 were selected for further study after preliminary experiments demonstrated that Tox-17 and Tox-18 failed to cause chlorotic symptoms in the sunflower bioassay. Subsequent cloning of regions of the Tox-17 and Tox-18 genomes containing the transposon insertions yielded fosmids pEP-T17-01 through -09 and pEP-T18-01 through -09, respectively. DNA sequence analysis indicated that the transposon was inserted in the region of the EB037 genome with a high degree of similarity to *gacS* in Tox-17 and *gacA* in Tox-18.

**Fig. 3:**
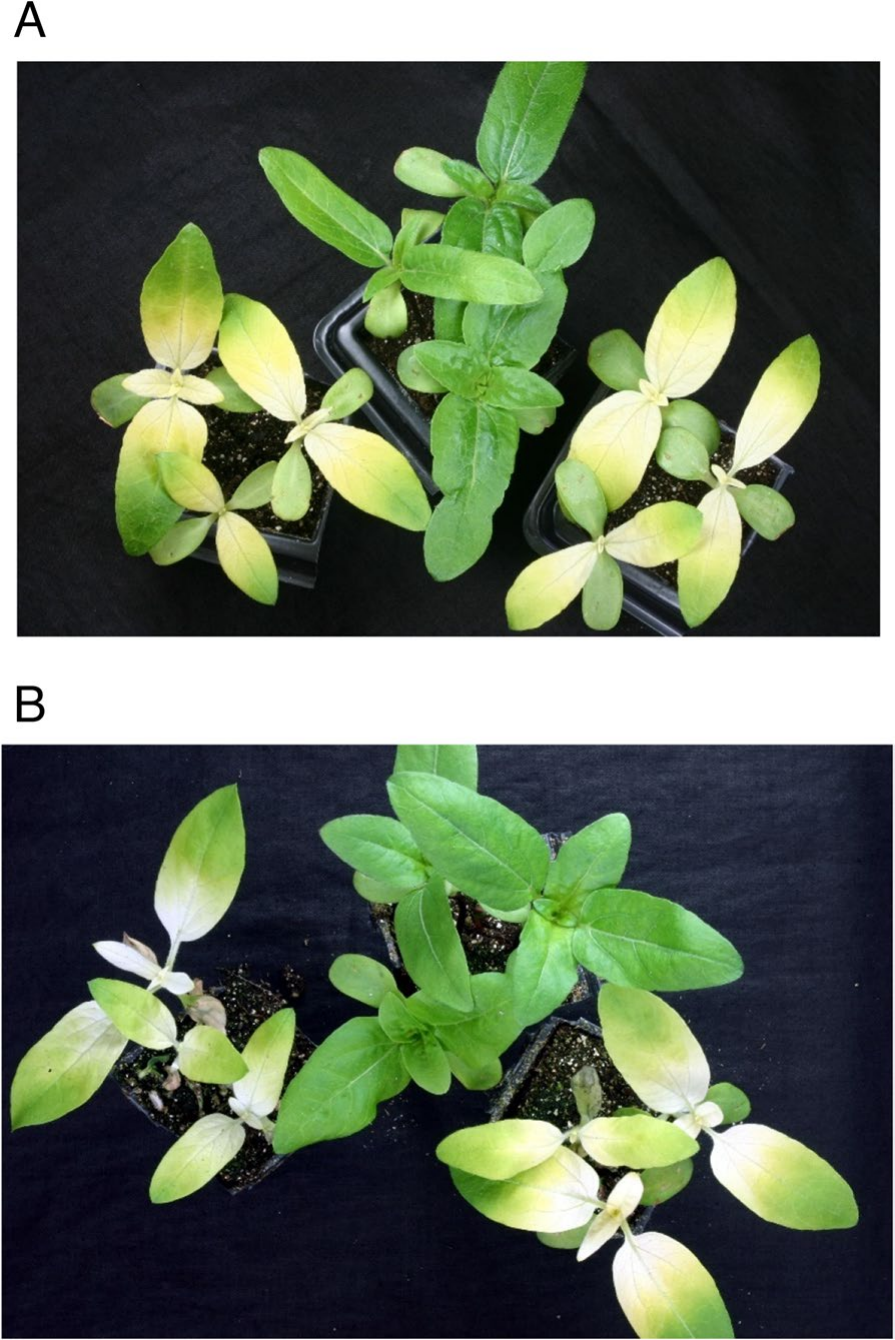
Sunflower bioassay for apical chlorosis. In each subfigure, sunflower plants were inoculated with cells of *Pseudomonas syringae* pv. *syringae* EB037 derivative strains just below the hypocotyle using a hypodermic needle. Plants were evaluated for apical chlorosis, indicative of tagetitoxin production, at 5 to 7 days after inoculation. **A** The leftmost plant was inoculated with EB037 containing the empty cloning vector pME6031, the center plant was inoculated with Tox-17, and rightmost plant was inoculated with Tox-17(pJLCP-11) which contains wild-type *gacS* in pME6031. **B** The leftmost plant was inoculated with EB037 containing the empty cloning vector pME6032, the center plant was inoculated with Tox-18, and rightmost plant was inoculated with Tox-18(pJLCP-5) which contains wild-type *gacA* in pME6032

The transposon insertion in *gacS* in Tox-17 resulted in a significant increase in the number of symptomless plants relative to the wild-type, EB037 in two experiments (Table 3). Addition of wild-type *gacS* to Tox-17 on plasmid pJLCP11 significantly reduced the number of symptomless plants relative to Tox-17 while the number of symptomless plants with the treatment containing Tox-17(pJLCP11) was like that obtained with the EB037 treatment. The photograph in Fig. 3 illustrates the loss of the pathogenicity phenotype associated with Tox-17 and restoration of the pathogencity phenotype associated with Tox-17(pJLCP11). Likewise, the transposon insertion in *gacA* in Tox-18 resulted in a significant increase in the number of symptomless plants relative to the wild-type, EB037 in two experiments (Supplementary Table S3). Addition of wild-type *gacA* to Tox-18 on plasmid pJLCP5 significantly reduced the number of symptomless plants relative to Tox-18 while the number of symptomless plants with the treatment containing Tox-18(pJLCP5) was similar with the EB037 treatment. The photograph in Fig. 3 shows the loss of the pathogenicity phenotype associated with Tox-18 and restoration of the pathogenicity phenotype associated with Tox-18(pJLCP5).

**Table 3:** Expression of chlorotic symptoms in sunflower inoculated with *Pseudomonas syringae* pv. *tagetis* Tox-17, containing a mutation in *gacS*, and derivative strains relative to the wild-type, EB037^a^

Treatment	Mean percent	Lower 95%	Upper 95%
	Symptomless plants	C.I.	C.I.
Experiment 1			
Tox-17	100 ± 0.0*	57.1	100
Tox-17(pJLCP11)	25.0 ± 12.5#	6.1	63.1
Tox-17(pME6031)	100 ± 0.0*	59.5	100
Tox-17(pME6032)	100 ± 0.0*	51.6	100
EB037	27.3 ± 13.4#	6.7	66.3
EB037(pME6031)	16.7 ± 10.8#	3.1	55.9
EB037(pME6032)	14.3 ± 13.2#	1.5	64.0
Experiment 2			
Tox-17	100 ± 0.0*	61.5	100
Tox-17(pJLCP11)	8.3 ± 8.0#	1.0	47.8
Tox-17(pME6031)	100 ± 0.0*	61.5	100
Tox-17(pME6032)	100 ± 0.0*	61.5	100
EB037	16.7 ± 10.8#	3.1	55.9
EB037(pME6031)	0.0 ± 0.0#	0.0	38.5
EB037(pME6032)	33.3 ± 13.6#	9.8	69.7

^a^For percent symptomless plants treatment values are the mean with standard error of twelve replicates (*n* = 12) from a single experiment.C.I., confidence interval

Tox17 was *P. syringae* pv. *tagetis* Tox17 while EB037 was the wildtype, *P. syringae* pv. *tagetis* EB037. Means followed by * were significantly different (*P* ≤ 0.05) in a pairwise comparison with the wild-type, EB037. Means followed by # were significantly different (*P* ≤ 0.05) in a pairwise comparison with Tox17

Twenty-two additional Rif- and Kan-resistant transposon mutant derivative strains of *Pstag* EB037 that failed to cause apical chlorosis on sunflower when injected into sunflower cotyledons in an initial screen were subjected to two replicate sunflower apical chlorosis disease assay experiments (Table 4). EB037 and Tox-17 served as the disease and loss of disease controls, respectively. All transposon mutant strains, except Tox-14, were significantly reduced in the ability to cause apical chlorosis relative to the wild-type, positive control EB037 in both disease assays (Table 4). Strain Tox-14 was significantly reduced relative to the positive control in the ability to cause apical chlorosis in one of two disease assays. All 22 mutant strains, including Tox-14, were similar with the loss of disease control, Tox-17, regarding the inability to cause apical chlorosis on sunflower in both disease assays. Tox-9 contains a mutation in *exbD*, detailed in a prior publication [59], but not previously rigorously tested for loss of ability to cause apical chlorosis on sunflower.

**Table 4:** Expression of chlorotic symptoms in sunflower inoculated with transposon mutants of *Pseudomonas syringae* pv. *tagetis* EB037^a^

	Experiment 1			Experiment 2		
Treatment	Mean percent	Lower 95%	Upper 95%	Mean percent	Lower 95%	Upper 95%
	Symptomless plants	C.I	C.I	Symptomless plants	C.I	C.I
Tox-1	100.0 ± 0.0*	59.5	100.0	100.0 ± 0.0*	61.5	100.00
Tox-3	100.0 ± 0.0*	61.5	100.0	100.0 ± 0.0*	61.5	100.00
Tox-4	100.0 ± 0.0*	61.5	100.0	100.0 ± 0.0*	61.5	100.00
Tox-5	100.0 ± 0.0*	61.5	100.0	100.0 ± 0.0*	61.5	100.00
Tox-6	100.0 ± 0.0*	61.5	100.0	100.0 ± 0.0*	61.5	100.00
Tox-17	100.0 ± 0.0*	61.5	100.0	100.0 ± 0.0*	61.5	100.00
EB037	0.0 ± 0.0#	0.0	38.5	0.0 ± 0.0#	0.0	38.5
Tox-7	100.0 ± 0.0*	61.5	100.0	100.0 ± 0.0*	54.5	100.0
Tox-8	100.0 ± 0.0*	59.5	100.0	100.0 ± 0.0*	54.5	100.0
Tox-9	100.0 ± 0.0*	59.5	100.0	100.0 ± 0.0*	54.5	100.0
Tox-11	100.0 ± 0.0*	59.5	100.0	100.0 ± 0.0*	54.5	100.0
Tox-17	100.0 ± 0.0*	61.5	100.0	100.0 ± 0.0*	61.5	100.0
EB037	0.0 ± 0.0#	0.0	40.5	0.0 ± 0.0#	0.0	45.5
Tox-12	100.0 ± 0.0*	54.5	100.0	100.0 ± 0.0*	61.5	100.00
Tox-13	100.0 ± 0.0*	51.6	100.0	100.0 ± 0.0*	61.5	100.00
Tox-14	62.5 0 ± 17.1	22.2	90.7	100.0 ± 0.0*	61.5	100.00
Tox-15	100.0 ± 0.0*	54.5	100.0	100.0 ± 0.0*	61.5	100.00
Tox-16	100.0 ± 0.0*	54.5	100.0	100.0 ± 0.0*	61.5	100.00
Tox-17	100.0 ± 0.0*	54.5	100.0	100.0 ± 0.0*	61.5	100.00
EB037	10.0 ± 0.0$	1.1	53.2	33.3 ± 13.6#	9.8	69.7
Tox-19	100.0 ± 0.0*	60.7	100.0	100.0 ± 0.0*	61.5	100.0
Tox-20	100.0 ± 0.0*	60.7	100.0	91.7 ± 8.0*	52.2	99.1
Tox-21	100.0 ± 0.0*	60.7	100.0	100.0 ± 0.0*	61.5	100.0
Tox-22	100.0 ± 0.0*	60.7	100.0	100.0 ± 0.0*	61.5	100.0
Tox-23	100.0 ± 0.0*	60.7	100.0	100.0 ± 0.0*	59.5	100.0
Tox-17	100.0 ± 0.0*	60.7	100.0	100.0 ± 0.0*	61.5	100.0
EB037	33.3 ± 13.6#	9.6	70.1	8.3 ± 8.0#	0.9	47.8
Tox-24	100.0 ± 0.0*	63.6	100.0	100.0 ± 0.0*	63.6	100.0
Tox-25	91.7 ± 8.0*	54.0	99.0	100.0 ± 0.0*	63.6	100.0
Tox-17	100.0 ± 0.0*	63.6	100.0	100.0 ± 0.0*	63.6	100.0
EB037	0.0 ± 0.0#	0.0	38.4	25.0 ± 12.5#	6.4	61.7

^a^For percent symptomless plants treatment values are the mean with standard error of twelve replicates (*n* = 12) from a single experiment. *C.I.* confidence interval. Tox-17, negative control, *P. syringae* pv. *tagetis* Tox-17. EB037, positive control, *P. syringae* pv. *tagetis* EB037. Means followed by * were significantly different (*P* ≤ 0.05) in a pairwise comparison with the wild-type, EB037. Means followed by # were significantly different (*P* ≤ 0.05) in a pairwise comparison with Tox17 and all other mutant strains within the group. Means followed by $ were significantly different (*P* ≤ 0.05) in a pairwise comparison with Tox17, Tox-12, Tox-13, Tox-15, and Tox-16

Genes containing the transposon insertion in all EB037 mutant derivative strains were subsequently identified (Table 5; Supplementary Table S4) and the strains verified to have single transposon insertions by whole genome sequencing. Transposon insertions within *gacS* (Tox-17, Tox-23, Tox-24), *gacA* (Tox-18), *hrcC* (Tox-25) of the TPI *hrp-hrc* gene cluster (Fig. 1), and *virD4* (Tox-11) of the T4SS (Fig. 2; Supplementary Table S1), and the resulting loss of apical chlorosis in disease assays correlated these genes and disease**.** Likewise, transposon insertions in Tox-3, Tox-4, and Tox-8 correlated an ATP-dependent DNA helicase, trehalose metabolism, and lytic murein transglucosylase, respectively, with disease. Mutants Tox-7, Tox-16, and Tox-22 had transposon insertions in genes appearing to function in general metabolism suggesting that the loss of disease symptoms may be due to fitness attenuation *in planta* [75]. A mutation in *raxQ* in Tox-20 resulted in loss of the ability of *Pstag* to cause apical chlorosis on sunflower. A mutation in this gene had the opposite effect on *Xanthomonas oryzae* in rice [109]. Mutational analysis identified genes correlated with apical chlorosis of sunflower (Tox-1, acetate kinase; Tox-5, *prlC*; Tox-6, cysteine synthase family protein; Tox-12, *putA*) that were also involved in disease in animal pathosystems. Three plasmid loci contained transposon insertions. Mutants Tox-6 and Tox-11, described above, as well as Tox-15, with an insertion in *xerD*, no longer caused apical chlorosis on sunflower*.* There was one transposon mutant, Tox-19, with an insertion in a gene with unknown identity/function and one mutant, Tox-14, where the transposon was inserted in an intergenic region of the genome.

**Table 5:** Transposon insertion sites in *Pseudomonas syringae* pv. *tagetis* EB037 derivative strains^a^

**Strain**	**Inactivated gene/protein**	**Description**
Tox-1	Acetate kinase	Acetate regulation was a factor in virulence of *Vibrio cholera* on *Drosophila melanogaster* [63]
Tox-3	UvrD-helicase domain-containing protein	An ATP-dependant DNA helicase influenced Diffusible Signal Factor (DSF) synthesis and transduction. DSF family molecules regulate diverse processes contributing to virulence of *Xanthomonas campestris* [90]
Tox-4	Trehalose transport system substrate binding protein SugABC	Synthesis and transport of trehalose from the cytosol into the periplasmic space was essential for osmotic control and pathogenicity of *Xanthomonas citri* subsp. *Citri* and *Pseudomonas aeruginosa* in plants. Trehalose uptake was essential for virulence of *Mycobacterium tuberculosis *[32, 34, 57]
Tox-5	*prlC*/Oligopeptidase A	Oligopeptidase A functions in degradation of signal peptides after release from precursor proteins. In *Haemophilus influenzae*, *prlC* was upregulated in biofilms [124]
Tox-6	Cysteine synthase family protein	Located within the plasmid portion of the *Pstag* EB037 genome. Inactivation of cysteine and methionine biosynthetic enzymes in *M. tuberculosis* significantly reduced persistence and virulence during infection in mice [37]
Tox-7	Histidinol dehydrogenase	Histidinol dehydrogenase catalyzes the terminal step in the biosynthesis of histidine. Mutation in *hisD* in *Pseudomonas savastanoi* resulted in reduced virulence on olive [75]
Tox-8	*mltB*, lytic murein transglycosylase	Involved in non-hydrolytic bacterial peptidoglycan depolymerization [28]. Mutation in *mltB* of *P. savastanoi* resulted in reduced virulence on olive [75]
Tox-9	*exbD*	Family of proteins containing ExbD were involved in TonB-dependent transport of various receptor bound substrates. A mutation in *exbD* in *P. syringae* pv. *tagetis* EB037 resulted in lost ability to cause apical chlorosis on sunflower [59]
Tox-10^b^	N-acetylglutaminyl glutamine amidotransferase	Glutamine amidotransferase (GATase) enzymes catalyzed the removal of the ammonia group from glutamine. N-acetylglutaminylglutamine amidotransferase is implicated in the synthesis of N-acetylglutaminylglutamine amide (NAGGN), a dipeptide necessary for osmoprotection [103]
Tox-11	*virD4*	Located within the plasmid portion of the *Pstag* EB037 genome. VirD4 forms the platform for T4SS substrate docking prior to secretion [41]. See Fig. 2. Flanked upstream by natural killer protein and hypothetical assistor proteins
Tox-12	*putA*	PutA was required for virulence of *P. aeruginosa*. A *putA* mutant was more susceptible to oxidative stress than the wild-type strain [135]
Tox-13	Putative phosphatase	Haloacid dehalogenase-like hydrolase
Tox-14	Intergenic region	Intergenic region between genes encoding a hypothetical protein and acyl-CoA dehydrogenase
Tox-15	*xerD*	Integrase within plasmid portion of the *Pstag* EB037 genome. Upstream and downstream genes encoded hypothetical proteins
Tox-16	*maeA*	*maeA* encodes a NAD-dependent malic enzyme (MaeA) which can function in gluconeogenesis. The gluconeogenic pathway for PEP synthesis was required for virulence of *X. campestris* pv. *campestris* (Xcc), and *Pseudomonas syringae* pv. *tomato.* In Xcc only the MaeA route was functional to synthesize PEP for gluconeogenesis and this gluconeogenic pathway was required for its full virulence [77, 115]
Tox-17, Tox-23, Tox-24	*gacS*	Signal transduction histidine-protein kinase GacS. Part of GacS/A TCS that positively controlled the production of homoserine lactones and the formation of the virulence factors in *P. syringae* pathovars [18, 52]
Tox-18	*gacA*	Response regulator GacA. Part of GacS/A TCS [18]
Tox-19	DUF2339 domain-containing protein	
Tox-20	*raxQ*	A mutation in *raxQ* resulted in increased virulence (hypervirulence) in *Xanthomonas oryzae* on rice [109]
Tox-21	Anthranilate synthase component 1	Part of a heterotetrameric complex that catalyzes the two-step biosynthesis of anthranilate, an intermediate in the biosynthesis of L-tryptophan
Tox-22	*ilvC*; ketol-acid reductoisomerase	Mutation in *ilvC* caused reduced virulence in *P. savastanoi* on olive [75]
Tox-25	*hrcC*	Component of T3SS [24]. See Fig. 1 for upstream and downstream genes

*T3SS* Type III secretion system, *T4SS* Type IV secretion system, *TCS* two-component system

^a^See Supplementary Table S4 for genome location, upstream genes, and downstream genes

^b^Tested for apical chlorosis with the initial screen only

## Discussion

*Pseudomonas syringae* pv. *tagetis* (*Pstag*) causes disease on sunflower and other plants of the Asteraceae family. To cause disease *Pstag* must overcome formidable plant defense systems, derive nutrients from host tissues, and aggressively increase in population in the plant apoplast. These plant defense systems include physical barriers, diverse arsenals of immune receptors capable of recognizing all classes of pathogens, and in some cases effectors. Plant responses include plasmodesmata and stomatal closure, callose deposition, production of reactive oxygen species (ROS), nitride oxide, phosphatidic acid, phytoalexins, and phytohormones, and programmed cell death (hypersensitive response) and systemic acquired resistance [117, 119, 131]. Our analysis of *Pstag* EB037 revealed a sophisticated repertoire of molecular mechanisms that potentially function in host plant defense evasion and disease.

As with other members of *P. syringae* Phylogroup 6, *Pstag* EB037 harbored a canonical TPI T3SS functioning in disease [1, 127]. With one exception, the sequence of genes and direction of transcription of the *hrp-hrc* gene cluster and CEL components of the TPI were identical between *Pstag* EB037 and *P. syringae* pv. *tomato* DC3000; the exception being that *cesT* replaced *schE* in the DC3000 CEL [1, 125]. The *Pstag* EB037 EEL did not contain identifiable effector genes, which is not unusual [127], but did have genes potentially functioning in disease or colonization of hostile environments (Fig. 1). SDR oxidase reductases and oxidoreductases in general have been shown to have roles in disease, colonization, and detoxification [96, 102, 118]. Glutathione reductase has a potential role in detoxification of harmful compounds [4] while *trxB* has a potential role in detoxifying ROS [53]. LysR-type transcriptional regulators and *tetR* family genes have been shown to be important for virulence of pathogenic bacteria of animals and plants [22, 72, 95, 133]. Two integrase genes were also detected within the EEL, consistent with the integron-like assembly of the EEL [17, 25].

Genome mining efforts detected homologues for 44 T3SS effector genes that were within the TPI or scattered throughout the *Pstag* EB037 genome (Table 2; Fig. 1). A core eight effectors were shown to be absolutely necessary for disease on *Arabidopsis* by DC3000 highlighting the importance of the roles that each of these eight effectors plays in disease [21, 85, 127]. Five of these effectors (AvrPtoB, HopG1, HopE1, HopAM1, HopN1) appear to be primarily involved in suppression of PTI and ETI. The role of two of the remaining core effectors, HopM1 and AvrE, appears to be counteracting inhibitory molecules produced due to PTI and ETI defense responses through induction of water soaking. Water soaking may reduce the concentration of inhibitory compounds in the apoplast and increase nutrients [127, 128]. *Pstag* EB037 has gene homologues for all 8 of these T3SS core effectors (Table 2).

Effectors not translocated through the T3SS likely contribute to disease through provision of parallel roles with T3SS effectors or additional roles. An example is tagetitoxin and the T3SS effector HopN1, and possibly HopK1 and HopO1-2; all targeting the host plant chloroplast. HopN1 was shown to localize to the plant chloroplast, degrade PsbQ and inhibit PSII activity [100]. HopK1 and HopO1-2 contain chloroplast transit peptides, but chloroplast targets are unknown [68]. Tagetitoxin targets chloroplast RNA polymerase resulting in plastids that have no, or poorly developed, thylakoid membranes. The combined effect of tagetitoxin and these T3SS effectors appears to be a debilitated chloroplast and in the plant apex no, or little, chloroplast formation. Chloroplasts make important contributions to plant defense through production of jasmonate and abscisic acid and possibly salicylic acid. The production of ROS and nitride oxide are also key defense contributions of chloroplast metabolism with the photosynthetic electron transport chains of PSI and PSII providing electrons for free radical formation [68].

Likewise, genes detected in the *Pstag* EB037 EEL (Fig. 1) such as *trxB* and those encoding SDR oxidase reductase and glutathione reductase may aid HopM1 and AvrE in enabling aggressive growth and persistence in the plant apoplast through detoxification of ROS and other inhibitory compounds. Additional *Pstag* EB037 genes detected by transposon mutagenesis may function in detoxification of the apoplast or persistence in unfavorable environments as well (Table 5). *putA* (Tox-12) was shown to be important in resisting oxidative stress by *P. aeruginosa* in an animal pathosystem. A trehalose transport system involving *sugABC* (Tox-4) was essential for osmotic control and pathogenicity *in planta* by *X. citri* subsp. *citri* and *P. aeruginosa* while N-acetylglutaminyl glutamine amidotransferase (Tox-10) was shown to be important for osmoprotection in plant- and animal-associated bacteria. *prlC* (Tox-5) was shown to be upregulated in biofilms in *H. influenzae.* Biofilms are known to protect bacterial populations in deleterious environments [12].

There is substantial evidence that horizontal gene transfer via conjugal plasmids and other mobile genetic elements played a significant role in evolution of virulence of *P. syringae* strains [6, 29, 30, 48, 70, 86, 106]. For example, most pathovars of *P. syringae* contain at least one indigenous plasmid, which typically encode determinants for ecological fitness or genes for Type III SS effectors or phytotoxins [70, 113, 114, 134]. Consistent with this, the *Pstag* EB037 genome contains at least two plasmids that contain loci important for apical chlorosis on sunflower; the pTagA-like plasmid having genes potentially functioning in conjugation.

Complete Class A T4SSs, such as the T4SS on the pTagA-like plasmid in *Pstag* EB037, and Class B T4SS have been detected on native plasmids of *P. syringae* pathovars as well as the detection of incomplete T4SS gene sets [6, 48, 94, 112, 134]. As with several bacterial human pathogens and the plant pathogens *Agrobacterium tumefaciens* and *Acidovorax oryzae* [41, 91], it appears that the *Pstag* EB037 T4SS functions in disease (Fig. 2; Table 4; Tox-11). No other strains of *P. syringae* have been reported to involve T4SSs in disease to our knowledge. Rather, T4SSs of plant pathogenic bacteria have typically been implicated in horizontal gene flow of virulence factors and as a killing machine to decrease competition with other bacteria within an ecological niche [48, 56, 111].

Candidates for translocation through the *Pstag* T4SS were hypothetical virulence genes associated with the *Pstag* T4SS locus and a killer protein homologue with a predicted function in ubiquination and cell apoptosis (Fig. 2). In animal pathosystems T4SSs transfer to eukaryotic target cells anywhere from one effector, like *Helicobacter pylori* delivering the effector CagA, to over 300 effector proteins as with *Legionella pneumophila* [41]. T4SS effector genes may be scattered throughout the genome as with the bacterial killing machine of *X. citri* [108], so it is possible that T4SS gene homologues are located elsewhere in the *Pstag* genome. There were no components of Type 6 Secretion Systems (T6SS) detected by our analysis of *Pstag* EB037 or any of the six types of secretion systems reported in various bacteria except for the T3SS and T4SS reported here. T6SSs have been reported to transport signaling molecules and toxic compounds and a T6SS was reported to be important in disease caused by *P. syringae* pv. *actinidiae* [42, 121].

## Conclusions

Apical chlorosis disease caused by *Pstag* EB037 is clearly the result of a complex set of mechanisms. This study identified homologues for at least 30 T3SS effectors with known functions in disease (Table 2) and another 20 genes in loci correlated with apical chlorosis on sunflower (Tables 3, 4, and 5). Going forward it is important to further our understanding of the interaction of *Pstag* EB037 T3SS effectors and these other genes during suppression of plant innate immunity and during growth and persistence in the apoplast environment, as well as the regulation of the involved genes. It is also important to further our understanding of the novel findings of this study; the role of the locus containing the *Pstag* EB037 T4SS and possible associated effector(s) in apical chlorosis disease. Further work with the 20 genes in loci associated with this study should provide further insights to the molecular basis of disease caused by *Pstag*.

## Materials and methods

### Bacterial strains and culture conditions

Descriptions of bacterial strains and plasmids used in this study are listed in Table 6. *Pstag* EB037 is a rifampicin (Rif) -resistant, tagetitoxin-producing strain that causes apical chlorosis on sunflower and other plants [59]. *Pstag* EB037 and EB037 derivative transposon mutant strains were routinely grown at 28^o^ C on King’s B (KB) agar or broth [58]. *Escherichia coli* EPI300, used in transposon mutagenesis, was obtained from the supplier (Epicentre Biotechnologies, Madison, WI) and routinely grown on Luria–Bertani (LB) broth or agar [80]. Where appropriate, media were supplemented with Rif at 50 µg ml^−1^, kanamycin (Kan) at 25 µg ml^−1^, or ampicillin (Ap) at 25 µg ml^−1^.

**Table 6:** Bacterial strains, plasmids, and primers

**Strain, plasmid, or primer**	**Relevant characteristics**^**a**^	**Reference or source**
Strains		
* P. syringae* pv. *tagetis* EB037	Wildtype, tagetitoxin-producing strain; Rif^R^	[59]
* Escherichia coli* EPI300	F^−^ *mcrA* Δ(*mrr-hsd*RMS*-mcr*BC) (Str^R^) Φ80d*lacZ*ΔM15 Δ*lacX74 rec*A1 *end*A1 *araD*13*9* Δ(*ara*, *leu*)7697 *galU galK* λ^−^ *rpsL nupG trfA ton*A *dhfr*	Epicentre Biotechnologies
* E. coli* DH5α	(Φ80d*lacZ*ΔM15) *Δ(lacZYA-argF*) *U169 glnV44 deoR gyrA96 recA1 relA91 endA1 thi-1 hsdR17*	
* E. coli* EC100	*F*^*−*^* mcrA Δ(mrr-hsdRMS-mcrBC) Φ80dlacZΔM15 ΔlacX74 recA1 endA1 araD139 Δ(ara, leu)7697 galU galK λ*^*−*^* rpsL (Str*^*R*^*) nupG*	Epicentre Biotechnologies
* E. coli* JM109	*endA1 glnV44 thi-1 relA1 gyrA96 recA1 mcrB*^+^ *Δ(lac-proAB) e14- [F' traD36 proAB*^+^ *lacI*^*q*^* lacZΔM15] hsdR17(r*_*K*_^*−*^*m*_*K*_^+^*)*	
* E. coli* TOP10	F- *mcrA* Δ(*mrr*-*hsdRMS*-*mcrBC*) Φ80*lacZ*ΔM15 Δ*lac*X74 *recA1* *araD139* Δ(*ara*-*leu*)7697 *galU* *galK* *rpsL* (StrR) *endA1* *nup*G	Invitrogen Corp
Plasmids		
pCR-XL-TOPO	Large PCR product cloning vector, Kan^R^	Invitrogen Corp
pCR4-TOPO	Cloning vector, Ap^R^, Kan^R^	Invitrogen Corp
pGEM-T	PCR product cloning vector; Ap^R^	Promega Corp
pRK2073	Helper plasmid, Tra^+^, Sm^R^	[31]
pCC1FOS	Fosmid cloning vector; Ap^R^	Epicentre Biotechnologies
pEP-T17-01 through -09	Fosmids containing DNA rescued from EB037 T17; Ap^R^	This study
pEP-T18-01 through -09	Fosmids containing DNA rescued from EB037 T17; Ap^R^	This study
pME6031	pVS1-p15A shuttle cloning vector for gram-negative bacteria; Tc^R^	[49]
pME6032	*lacI*^q^- P_tac_ expression vector derived from pME6031; Tc^R^	[49]
pJLCP-4	Contains WT *gacA* from EB037 cloned in pGEM-T, Ap^R^	This study
pJLCP-5	10.45 kb; contains WT *gacA* from EB037 cloned in pME6032; Tc^R^	This study
pJLCP-10	6.83 kb; contains WT *gacS *and promoter from B728a cloned in pCR-XL-TOPO; Kan^R^	This study
pJLCP-11	11.64 kb; Contains WT *gacS* from B728a cloned in pME6031; Tc^R^	This study
Primers		
GACA-F2/GACA-R1	5'-ACACGAATTCTATGACCGTTGCGCGAGG/ 5'-ACACCTCGAGTCAGGCGCTGGCATC	This study
GACS-F19/GACS-R19	5'-AATCATCCGGTAGCCCTTG/ TGCTGTAGCGAAAATCGTGT	This study
RSMA-F10/RSMA-R10	5'-CTCGAATTCATGCTGATTCTGACTCG/ 5'-ATACTCGAGTTAATGGCTTGGTTC	This study
RSME-F1/RSME-R1	5'-CTGAATTCATGTTGATACTCACTCG/	This study
TAGTOX-17-FP1/TAGTOX-17-RP1	5'-CTCTCGAGTTATTGTGGGTCCT 5'-GAAATTCTCGACTTCTCGAAGATCG/ 5'-AATATTGAGCGATTCTTCCTTGTCTTC	This study
TAGTOX-18-FP1/TAGTOX-18-RP1	5'-TTTCGCCGGTCAACGTTACAT/ 5'-GCGTTATCGGACAGAAACACATAGG	This study

^a^*Ap*^*R*^ ampicillin-resistant, *Kan*^*R*^ kanamycin-resistant, *Rif*^*R*^ rifampicin-resistant, *Tc*^*R*^ tetracycline-resistant

### Genome sequencing and gene discovery

Strain EB037 and the derivative transposon mutants that failed to produce chlorotic symptoms in the sunflower prescreen (see below) were selected for DNA sequencing. Preparations of DNA were made using the Blood & Cell Culture DNA Miniprep kit as per the manufacturer’s instructions (Qiagen, Valencia, CA). Sequence data were generated on four lanes of an Illimina NextSeq-500 using the run kit Illumina NextSeq® 500/550 High Output Kit v2. The indexed library was constructed using Nextera® XT Index Kit v2 Set A. Sequencing resulted in 7.8 million reads with a mean read length of 147 bp, which comprised a total of 1.16 billion bases. All computation and analysis of data were done through Linux for Windows in a Ubuntu 18.04 LTS terminal. The suite of programs and scripts were available on GitHub (github.org). Sequence assembly was conducted with a paired-end Spades version 3.5.0 assembly pipeline using python version 2.7.15, and used standard k-mer sizes of 21, 33, and 55. Assemblies were run in Multi-cell mode with no coverage cutoff and with repeat resolution enabled. Analysis of the assemblies was conducted by QUAST (Quality Assessment Tool for Genome Assemblies version 5.0.2) against a reference genome (*P. syringae* pv. *syringae* B278a). The QUAST (Quality Assessment Tool for Genome Assemblies) algorithm [47] was used to determine that the assemblies had a score of > 35. The draft genome of *Pstag* EB037 was constructed using 94 contigs united into 10 scaffolds with a NGA50 of 69,652 and a LGA50 of 22. Assemblies were annotated with prokka version 1.13.7 (Bioperl version 1.007002) using packages blastp version 2.9, makeblast version 2.9, and hmmpress/hmmscan version 3.1. Genome features were functionally annotated using the algorithms Kmers V2 and protein similarity *P. syringae*. In total 5580 features were called, of which 68 were non-coding and 3540 were genes with functional predictions.

The annotated genome was then analyzed using Artemis version 18.0.3 under standard shell parameters. In cases when ORFs in operon-like regions were not annotated, we conducted a manual annotation using homology comparisons and three-dimensional protein prediction for analysis of proteins of interest with the web application- i-Tasser [130]. Similarity between protein structures were characterized by Gene Ontology (GO) c-scores. Disease-related genes were identified through a combination of keyword and homology searches of the EB037 draft genome in Artemis. Genomic BLAST was used to confirm gene identities and operon boundaries [5]. For each of the derivative transposon mutants, insertion sites were identified by scanning each genome assembly using homology searches to the transposon Kan^R^ gene which is inserted in the derivative mutant genome during mutagenesis. Dot plots were conducted using MegAlign in the DNAstar suite of software (ver. 15.1) from Lasergene® (Madison, WI). Genomes were compared pairwise relying on a minimum match of 50% and a window size of 30 and a minimum number of windows set at 1. Genomes were submitted to GenBank (SubmissionID: SUB8232311; BioProject ID: PRJNA1004645).

### Transposon mutagenesis and selection of apical chlorosis-deficient mutants

Mutants of Rif-resistant *Pstag* EB037 were generated by transposon mutagenesis as described by Kong et al. [59] with the EZ::TN™ < KAN-2 > Transposon kit following the manufacturer’s instructions (Epicentre Biotechnologies). Briefly, the EZ::TN < KAN-2 > transposon – EZ::TN transposase complex was introduced into strain EB037 by electroporation (25 μF, 2.5 kV, and 200 Ω with a field strength of 12.5 kV cm^−1^ for 4.5 to 5 ms) followed by plating on KB agar containing Rif and Kan. Rif- and Kan-resistant colonies (5594 colonies) were picked and stabbed into 7-day-old sunflower plants and the plants observed for chlorosis as described below for the sunflower bioassay. Colonies resulting in intermediate phenotypes in the sunflower bioassay were rescreened. Of 5594 Rif- and Kan- resistant colonies screened in planta, 24 consistently failed to produce chlorotic symptoms on sunflower. Growth of mutants were compared with that of the wild-type strain, EB037 on KB agar and LB agar.

### Transposon mutant complementation analysis

Preparations of genomic and plasmid DNA were made using the Blood & Cell Culture DNA and QIAprep Spin Miniprep kits, respectively, according to the manufacturer’s instructions (Qiagen, Valencia, CA). Restriction enzyme digestion, ligation, electrophoresis, and transformation procedures were performed using standard protocols. Electroporation was essentially as described where the electroporation apparatus was set to deliver an electrical pulse of 25 μF, 2.5 kV, and 200 Ω with a field strength of 12.5 kV/cm for 4.5 to 5 ms. Oligonucleotide primers (Table 6) were designed using Primer3 software at the Primer3 Web site (http://frodo.wi.mit.edu/cgi-bin/primer3/primer3_www.cgi) and synthesized by Invitrogen (Grand Island, N.Y.).

Genomic DNA was extracted from transposon mutants that failed to produce chlorotic symptoms in the sunflower prescreen and partially digested with *Eco*RV. Partially digested genomic DNA was separated on 1% agorose gels, and the band area representing approximately 40 kb cloned using the CopyControl™ Fosmid Library Production Kit (Epicentre Biotechnologies). Purified fosmid DNA containing DNA rescued from mutants EB037 T17 (fosmids pEP-T17-01 through -09) and EB037 T18 (fosmids pEP-T18-01 through -09) were shown to contain single EZ::TN™ < KAN-2 > insertions within *gacS* and *gacA*, respectively, by DNA sequencing using the FP1 and RP1 sequencing primers from the transposon kit (Epicentre Biotechnologies). Mutants Tox-17 and Tox-18 were confirmed to contain mutations in *gacS* and *gacA* by DNA sequencing using primers TAGTOX-17-FP1/TAGTOX-17-RP1 and TAGTOX-18-FP1/TAGTOX-18-RP1, respectively. DNA sequencing for complementation analysis was performed at the Centre for Biosystems Research DNA Sequencing Facility, Univ. of Maryland, College Park, MD on an ABI DNA sequencer (Applied Biosystems, Foster City, CA).

For construction of pJLCP-5, *gacA* plus 25 bp of upstream DNA was amplified from genomic DNA from the wild-type strain, EB037, using primers GACA-F2 and GACA-R1. These primers were designed based on sequence obtained from the mutated *gacA* gene in Tox-18. The PCR fragment was ligated to pGEM-T (Promega Corp., Madison, WI) creating pJLCP-4 and transformed into *E. coli* JM109. Plasmid pJLCP-4 was digested with *Eco*RI and *Xho*I, and the resulting 676-bp DNA fragment ligated to pME6032 that had been previously digested with *Eco*RI and *Xho*I. This ligation mixture was transformed into *E. coli* EC100 and *E. coli* colonies containing pJLCP-5 selected on KB plus Tc. pJLCP-5 construction was verified by sequencing and by restoration of Tox-18 chlorotic symptom production in the sunflower bioassay after mobilization of pJLCP-5 into Tox-18 by triparental mating with EC100(pJLCP-5) and *E. coli* DH5α(pRK2073). For construction of pJLCP-11, elongase PCR was used to amplify *gacS* and 526 bp of upstream DNA from *P. syringae* pv. *syringae* B728a genomic DNA using primers GACS-F19 and GACS-R19. The resulting 3.33-kb amplicon was digested with *Nco*I, ligated to pCR-XL-TOPO, and transformed into *E. coli* TOP10 (Invitrogen Corp., Carlsbad, CA). The resulting plasmid, pJLCP-10, was digested with *Nco*I and the 3.33-kb DNA fragment containing wildtype *gacS* and promoter was ligated to the promoter-less shuttle vector pME6031, which had been previously digested with *Nco*I, to create pJLCP-11. This ligation mixture was transformed into *E. coli* EC100 and *E. coli* EC100 colonies containing pJLCP-11 selected on KB agar plus Tc. pJLCP-11 construction was verified by DNA sequencing and by restoration of EB037 Tox-17 chlorotic symptom production in the sunflower bioassay following electroporation of EB037 Tox-17 with this construct.

## Sunflower bioassay

A sunflower (*Helianthus annuus* L. cv. ‘Autumn Beauty’) bioassay for the detection of chlorotic symptoms was performed essentially as described [45, 59]. Plants were grown in 50-cell flats containing Jiffy Mix Plus (Jiffy Products of America, Batavia, IL) at 24^o^ C in the growth chamber with a 16 h photoperiod (photosynthetic photon flux density of 600 μmol m^−2^ s^−1^). After 7 days, bacterial cells lifted from agar plates were inoculated into the plant just below the cotyledons by stabbing the stem with a 25-gauge hypodermic needle and plants returned to the growth chamber. Plants were evaluated visually for apical chlorosis at 5 to 7 days after injection. Experiments were performed twice with twelve replicate plants per treatment.

Lower and upper 95% confidence intervals were calculated for the number of symptomless plants for each treatment using Wilson’s scoring method for individual binomial proportions [87] and for differences between two binomial proportions [88]. To attain an experimentwise α = 0.05, a Sidak-adjusted comparisonwise α = [1 – (1—0.05)**(1/7)] = 0.0073 was used for each of the individual treatment proportions and α = [1 – (1 – 0.05)**(1/21)] = 0.0024 was used for each of their pairwise comparisons. Computations were accomplished using Base SAS version 9.4 (SAS Institute, Cary, NC). Statistical difference was determined by the confidence interval for the difference in treatment pair’s proportions does not contain zero. The standard errors were calculated using the formula for the standard error of a binomial distribution. Data from experiments were analyzed independently.

## Supplementary Information

Additional file 1: Supplementary Tables S1. *Pstag* EB037 pTagA-Like Plasmid Annotation. S2. *Pstag* EB037 pTagB-Like Plasmid Annotation. S3. Expression of Chlorotic Symptoms in Sunflower Inoculated with *Pseudomonas syringae* pv. *tagetis *Tox-18, Containing a Mutation in *gacA*, and Derivative Strains Relative to the Wild-Type, EB037; and S4. Tox Mutants: Genome Location of Transposon Insertion, Upstream Genes, and Downstream Genes and Supplementary Figs. S1. Dot Plot Analyses and S2. Image of Sunflower Bioassay.

## Data Availability

Sequence data that support the findings of this study were submitted to GenBank (SubmissionID: SUB8232311; BioProject ID: PRJNA1004645).

## Abbreviations

Ap: Ampicillin
CEL: Conserved effector locus
EEL: Exchangeable effector locus
EPS: Exopolysaccharide
ETI: Effector-triggered immunity
GO: Genome ontology
Kan: Kanamycin
LB: Luria-Bertani
PAMP: Pathogen associated molecular pattern
*Pstag*: *Pseudomonas syringae* pv. *tagetis*
PTI: PAMP—triggered immunity
Rif: Rifampicin
ROS: Reactive oxygen species
T3SS: Type III secretion system
T4SS: Type IV secretion system
T6SS: Type VI secretion system
TPI: Tripartite pathogenicity island
