# Supplementary material for: Genomic and mutational analysis of Pseudomonas syringae pv. tagetis EB037 pathogenicity on sunflower
Source: BMC Microbiol. 2025 Jan 24;25:43. doi: 10.1186/s12866-024-03685-8 (PMC11760712; doi:10.1186/s12866-024-03685-8)
Supplement: Supplementary file 1 — Additional file 1: Supplementary Tables S1. Pstag EB037 pTagA-Like Plasmid Annotation. S2. Pstag EB037 pTagB-Like Plasmid Annotation. S3. Expression of Chlorotic Symptoms in Sunflower Inoculated with Pseudomonas syringae pv. tagetis Tox-18, Containing a Mutation in gacA, and Derivative Strains Relative to the Wild-Type, EB037; and S4. Tox Mutants: Genome Location of Transposon Insertion, Upstream Genes, and Downstream Genes and Supplementary Figs. S1. Dot Plot Analyses and S2. Image of Sunflower Bioassay. [file 12866_2024_3685_MOESM1_ESM.docx]

| **Supplementary Table S1. Pstag EB037 pTagA-Like Plasmid Annotation** | | | | | | |
| --- | --- | --- | --- | --- | --- | --- |
|  |  |  |  |  |  |  |
| **Name** | **Minimum** | **Maximum** | **Length** | **Direction** |  |  |
| hyp4-vir | 84 | 326 | 243 | reverse |  |  |
| rfa3A | 416 | 949 | 534 | reverse |  |  |
| hyp6-vir | 1555 | 1737 | 183 | reverse |  |  |
| proP 7 CDS | 1800 | 3146 | 1347 | reverse |  |  |
| lhgO CDS | 3183 | 4379 | 1197 | reverse |  |  |
| lutR 6 CDS | 4443 | 5144 | 702 | reverse |  |  |
| xerC 9 CDS | 5407 | 6369 | 963 | forward |  |  |
| hyp7-vir | 6439 | 6759 | 321 | reverse |  |  |
| hyp8-vir | 7225 | 7548 | 324 | reverse |  |  |
| virC1 CDS | 7541 | 8239 | 699 | reverse |  |  |
| hypothetical protein CDS | 8492 | 8914 | 423 | forward |  |  |
| hypothetical protein CDS | 9363 | 9596 | 234 | forward |  |  |
| hypothetical protein CDS | 9625 | 9861 | 237 | forward |  |  |
| hypothetical protein CDS | 9861 | 10076 | 216 | forward |  |  |
| hypothetical protein CDS | 10508 | 10888 | 381 | forward |  |  |
| hypothetical protein CDS | 10908 | 11219 | 312 | reverse |  |  |
| hypothetical protein CDS | 11627 | 12001 | 375 | forward |  |  |
| hypothetical protein CDS | 12620 | 12982 | 363 | forward |  |  |
| csrA 2 CDS | 13248 | 13457 | 210 | reverse |  |  |
| hypothetical protein CDS | 13499 | 13804 | 306 | reverse |  |  |
| hypothetical protein CDS | 13838 | 14164 | 327 | reverse |  |  |
| hypothetical protein CDS | 14176 | 15255 | 1080 | reverse |  |  |
| hypothetical protein CDS | 15255 | 17051 | 1797 | reverse |  |  |
| umuC 3 CDS | 17214 | 18476 | 1263 | reverse |  |  |
| umuD 4 CDS | 18490 | 18915 | 426 | reverse |  |  |
| hypothetical protein CDS | 19054 | 20367 | 1314 | reverse |  |  |
| hypothetical protein CDS | 20765 | 20992 | 228 | reverse |  |  |
| hypothetical protein CDS | 21049 | 21498 | 450 | reverse |  |  |
| hypothetical protein CDS | 21675 | 21767 | 93 | reverse |  |  |
| hypothetical protein CDS | 21845 | 22090 | 246 | reverse |  |  |
| hypothetical protein CDS | 22222 | 24561 | 2340 | reverse |  |  |
| hypothetical protein CDS | 24876 | 25295 | 420 | reverse |  |  |
| hypothetical protein CDS | 25432 | 25743 | 312 | forward |  |  |
| hypothetical protein CDS | 25918 | 26328 | 411 | forward |  |  |
| hypothetical protein CDS | 26279 | 26626 | 348 | reverse |  |  |
| hypothetical protein CDS | 26863 | 27381 | 519 | forward |  |  |
| hypothetical protein CDS | 27408 | 27980 | 573 | reverse |  |  |
| hypothetical protein CDS | 27977 | 28711 | 735 | reverse |  |  |
| hypothetical protein CDS | 29042 | 29293 | 252 | reverse |  |  |
| hypothetical protein CDS | 29275 | 30207 | 933 | reverse |  |  |
| hypothetical protein CDS | 30191 | 30445 | 255 | reverse |  |  |
| ssb 2 CDS | 30525 | 31130 | 606 | reverse |  |  |
| hyp1-vir | 31158 | 31442 | 285 | reverse |  |  |
| topB 5 CDS | 31471 | 33636 | 2166 | reverse |  |  |
| killer protein | 33664 | 34026 | 363 | reverse |  |  |
| virD4 | 34067 | 35719 | 1653 | reverse |  |  |
| hyp2-vir | 35719 | 35949 | 231 | reverse |  |  |
| hyp3-vir | 36139 | 36495 | 357 | reverse |  |  |
| virB11 | 36505 | 37536 | 1032 | reverse |  |  |
| virB10 | 37546 | 38913 | 1368 | reverse |  |  |
| virB9 | 38900 | 39709 | 810 | reverse |  |  |
| virB8 CDS | 39699 | 40487 | 789 | reverse |  |  |
| virB7 | 40525 | 40827 | 303 | reverse |  |  |
| virB6 | 40895 | 41827 | 933 | reverse |  |  |
| hyp4-vir | 41838 | 42191 | 354 | reverse |  |  |
| virB5 | 42225 | 42911 | 687 | reverse |  |  |
| virB4 CDS | 42914 | 45460 | 2547 | reverse |  |  |
| virB3 | 45810 | 46145 | 336 | reverse |  |  |
| virB2 | 46145 | 46852 | 708 | reverse |  |  |
| virB1 | 46865 | 47107 | 243 | reverse |  |  |
|  |  |  |  |  |  |  |
|  |  |  |  |  |  |  |
|  |  |  |  |  |  |  |
|  |  |  |  |  |  |  |

| **Supplementary Table S2. Pstag EB037 pTagB-Like Plasmid Annotation** | |  |  |  |  |
| --- | --- | --- | --- | --- | --- |
|  |  |  |  |  |  |
| **Name** | **Type** | **Minimum** | **Maximum** | **Length** | **Direction** |
| xerD 2 CDS | CDS | 33 | 650 | 618 | forward |
| hypothetical protein CDS | CDS | 1148 | 1249 | 102 | forward |
| hypothetical protein CDS | CDS | 1588 | 2163 | 576 | reverse |
| hypothetical protein CDS | CDS | 2351 | 2992 | 642 | reverse |
| hypothetical protein CDS | CDS | 3204 | 3491 | 288 | reverse |
| hypothetical protein CDS | CDS | 3481 | 4131 | 651 | reverse |
| hypothetical protein CDS | CDS | 4244 | 4444 | 201 | reverse |
| hypothetical protein CDS | CDS | 4537 | 4698 | 162 | reverse |
| hypothetical protein CDS | CDS | 4782 | 5192 | 411 | reverse |
| hypothetical protein CDS | CDS | 5286 | 5573 | 288 | reverse |
| hypothetical protein CDS | CDS | 5839 | 6159 | 321 | forward |
| umuC 1 CDS | CDS | 6235 | 7533 | 1299 | reverse |
| umuD 2 CDS | CDS | 7511 | 7936 | 426 | reverse |
| hypothetical protein CDS | CDS | 8075 | 9391 | 1317 | reverse |
| hypothetical protein CDS | CDS | 9957 | 10310 | 354 | reverse |
| hypothetical protein CDS | CDS | 10301 | 10951 | 651 | reverse |
| hypothetical protein CDS | CDS | 11081 | 14830 | 3750 | reverse |
| hypothetical protein CDS | CDS | 14820 | 15545 | 726 | reverse |
| hypothetical protein CDS | CDS | 15770 | 16309 | 540 | forward |
| hypothetical protein CDS | CDS | 16332 | 16904 | 573 | reverse |
| hypothetical protein CDS | CDS | 16901 | 17620 | 720 | reverse |
| Single-stranded DNA-binding protein CDS | CDS | 17928 | 18533 | 606 | reverse |
| hypothetical protein CDS | CDS | 18597 | 19364 | 768 | reverse |
| topB 4 CDS | CDS | 19392 | 21557 | 2166 | reverse |
| hypothetical protein CDS | CDS | 21585 | 21947 | 363 | reverse |
| hypothetical protein CDS | CDS | 21988 | 23649 | 1662 | reverse |
| hypothetical protein CDS | CDS | 23649 | 23879 | 231 | reverse |
| hypothetical protein CDS | CDS | 24006 | 24362 | 357 | reverse |
| Type IV secretion system protein VirB11 CDS | CDS | 24372 | 25403 | 1032 | reverse |
| hypothetical protein CDS | CDS | 25413 | 26780 | 1368 | reverse |
| ptlF CDS | CDS | 26767 | 27576 | 810 | reverse |
| hypothetical protein CDS | CDS | 27566 | 28354 | 789 | reverse |
| hypothetical protein CDS | CDS | 28736 | 29680 | 945 | reverse |
| hypothetical protein CDS | CDS | 29707 | 29913 | 207 | reverse |
| hypothetical protein CDS | CDS | 29929 | 30609 | 681 | reverse |
| hypothetical protein CDS | CDS | 30606 | 33080 | 2475 | reverse |
| hypothetical protein CDS | CDS | 33019 | 33495 | 477 | reverse |
| hypothetical protein CDS | CDS | 33508 | 33843 | 336 | reverse |
| virB1 CDS | CDS | 33843 | 34550 | 708 | reverse |
| hypothetical protein CDS | CDS | 34563 | 34805 | 243 | reverse |
| rfaH 2 CDS | CDS | 34894 | 35478 | 585 | reverse |
| hypothetical protein CDS | CDS | 35623 | 36018 | 396 | forward |
| hypothetical protein CDS | CDS | 36030 | 36227 | 198 | reverse |
| potF CDS | CDS | 36325 | 37425 | 1101 | reverse |
| malT 2 CDS | CDS | 37534 | 38307 | 774 | forward |
| hypothetical protein CDS | CDS | 38423 | 38773 | 351 | reverse |
| hypothetical protein CDS | CDS | 38786 | 39034 | 249 | reverse |
| xerC 6 CDS | CDS | 39372 | 40334 | 963 | forward |
| hypothetical protein CDS | CDS | 41049 | 41429 | 381 | reverse |
| hypothetical protein CDS | CDS | 41759 | 42751 | 993 | reverse |
| cysL 2 CDS | CDS | 42909 | 43805 | 897 | reverse |
| hypothetical protein CDS | CDS | 43923 | 45092 | 1170 | forward |
| hypothetical protein CDS | CDS | 45101 | 45703 | 603 | reverse |
| ppa 2 CDS | CDS | 45703 | 46254 | 552 | reverse |
| eno 2 CDS | CDS | 46364 | 47647 | 1284 | reverse |
| hypothetical protein CDS | CDS | 47721 | 48062 | 342 | reverse |
| hypothetical protein CDS | CDS | 48157 | 48423 | 267 | reverse |
| hypothetical protein CDS | CDS | 48466 | 48789 | 324 | reverse |
| hypothetical protein CDS | CDS | 48870 | 49079 | 210 | reverse |
| eriC CDS | CDS | 49232 | 50578 | 1347 | reverse |
| hypothetical protein CDS | CDS | 50827 | 51150 | 324 | reverse |
| hypothetical protein CDS | CDS | 51161 | 51904 | 744 | reverse |
| hypothetical protein CDS | CDS | 52864 | 53088 | 225 | reverse |
| hypothetical protein CDS | CDS | 53772 | 55391 | 1620 | reverse |
| hypothetical protein CDS | CDS | 55584 | 56267 | 684 | forward |
| xerC 7 CDS | CDS | 56269 | 56895 | 627 | forward |
| xerD 3 CDS | CDS | 56943 | 57197 | 255 | forward |
| hypothetical protein CDS | CDS | 57610 | 57699 | 90 | forward |
| pglA CDS | CDS | 58291 | 59889 | 1599 | reverse |
| nadA CDS | CDS | 60285 | 61343 | 1059 | forward |
| bepA 4 CDS | CDS | 61395 | 62828 | 1434 | reverse |
| tusA 2 CDS | CDS | 62969 | 63220 | 252 | forward |
| hypothetical protein CDS | CDS | 63257 | 64327 | 1071 | forward |
| bcp CDS | CDS | 64382 | 64855 | 474 | reverse |
| gcvR CDS | CDS | 64865 | 65428 | 564 | reverse |
| dapA 1 CDS | CDS | 65754 | 66632 | 879 | forward |
| bamC CDS | CDS | 66651 | 67769 | 1119 | forward |
| yycJ CDS | CDS | 67770 | 68528 | 759 | forward |
| purC CDS | CDS | 68557 | 69267 | 711 | forward |
| tRNA-Ser | tRNA | 69359 | 69448 | 90 | forward |
| hypothetical protein CDS | CDS | 69567 | 70055 | 489 | reverse |
| hypothetical protein CDS | CDS | 70074 | 70346 | 273 | reverse |
| hypothetical protein CDS | CDS | 71665 | 71919 | 255 | forward |
| hypothetical protein CDS | CDS | 71922 | 72236 | 315 | forward |
| hypothetical protein CDS | CDS | 72489 | 72896 | 408 | forward |

Supplementary TABLE 3. Expression of chlorotic symptoms in sunflower inoculated with *Pseudomonas syringae* pv. *tagetis* Tox-18, containing a mutation in *gacA*, and derivative strains relative to the wild-type, EB037^a^

Treatment Mean percent Lower 95% Upper 95%

symptomless plants C.I. C.I.
­­­­­­­­­­­­­­­­­­______________________________________________________________________________

Experiment 1

Tox-18 100 + 0.0* 63.6 100

Tox-18(pJLCP5) 25.0 + 12.5# 6.4 61.7

Tox-18(pME6032) 100 + 0.0* 63.6 100

EB037 8.3 + 8.0# 1.0 46.0

EB037(pME6032) 0.0 + 0.0# 0.0 36.4

Experiment 2

Tox-18 100 + 0.0* 63.6 100

Tox-18(pJLCP5) 33.3 + 13.6# 10.3 68.5

Tox-18(pME6032) 100 + 0.0* 63.6 100

EB037 8.3 + 8.0# 1.0 46.0

EB037(pME6032) 0.00 + 0.0# 0.0 36.4

^a^For percent symptomless plants treatment values are the mean with standard error of twelve replicates (n = 12) from a single experiment. C.I., confidence interval. Tox-18 was *P. syringae* pv. *tagetis* Tox-18 while EB037 was the wildtype, *P. syringae* pv. *tagetis* EB037. Means followed by * were significantly different (*P* < 0.05) in a pairwise comparison with the wild-type, EB037. Means followed by # were significantly different (*P* < 0.05) in a pairwise comparison with Tox-18.


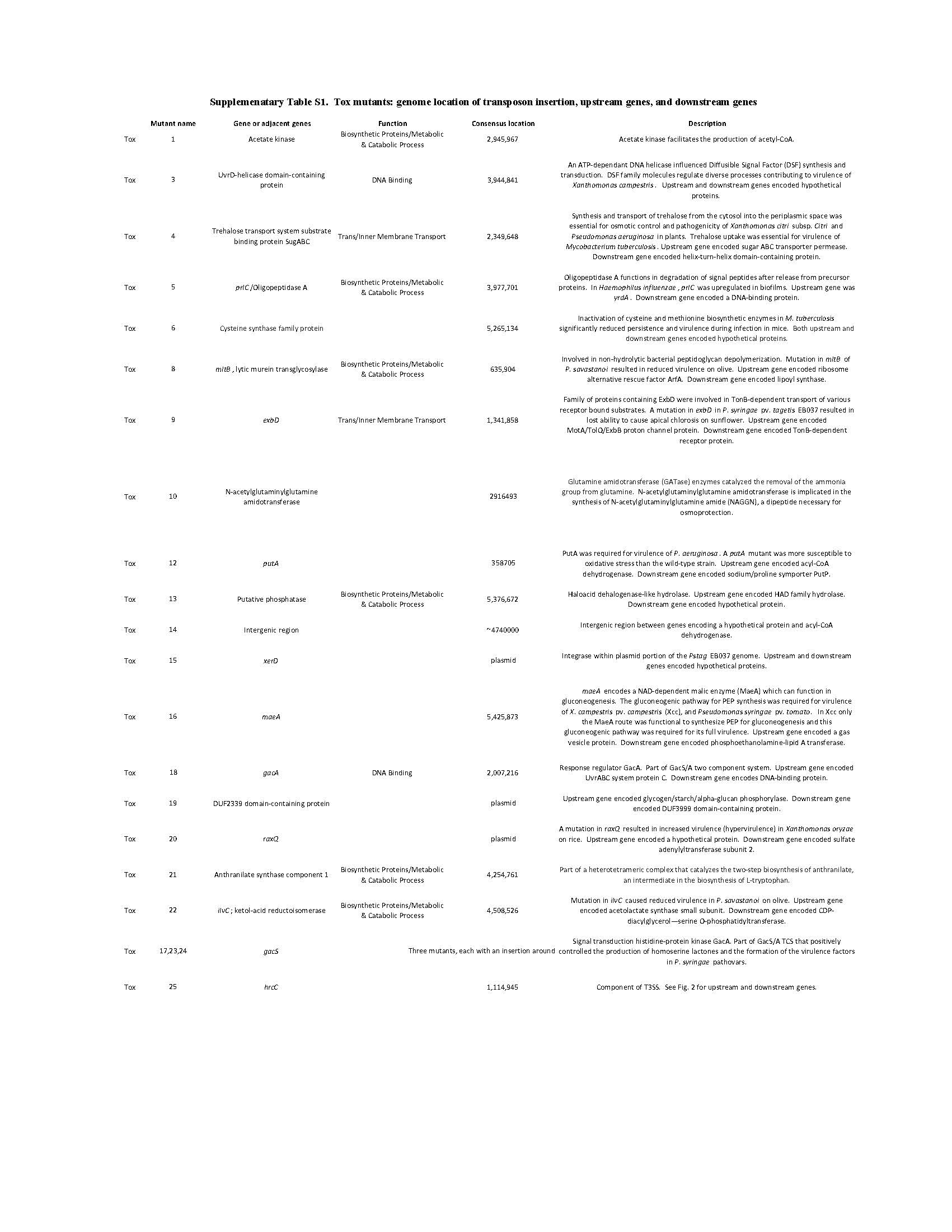


**Supplementary Fig S1: Dot Plot Analyses**


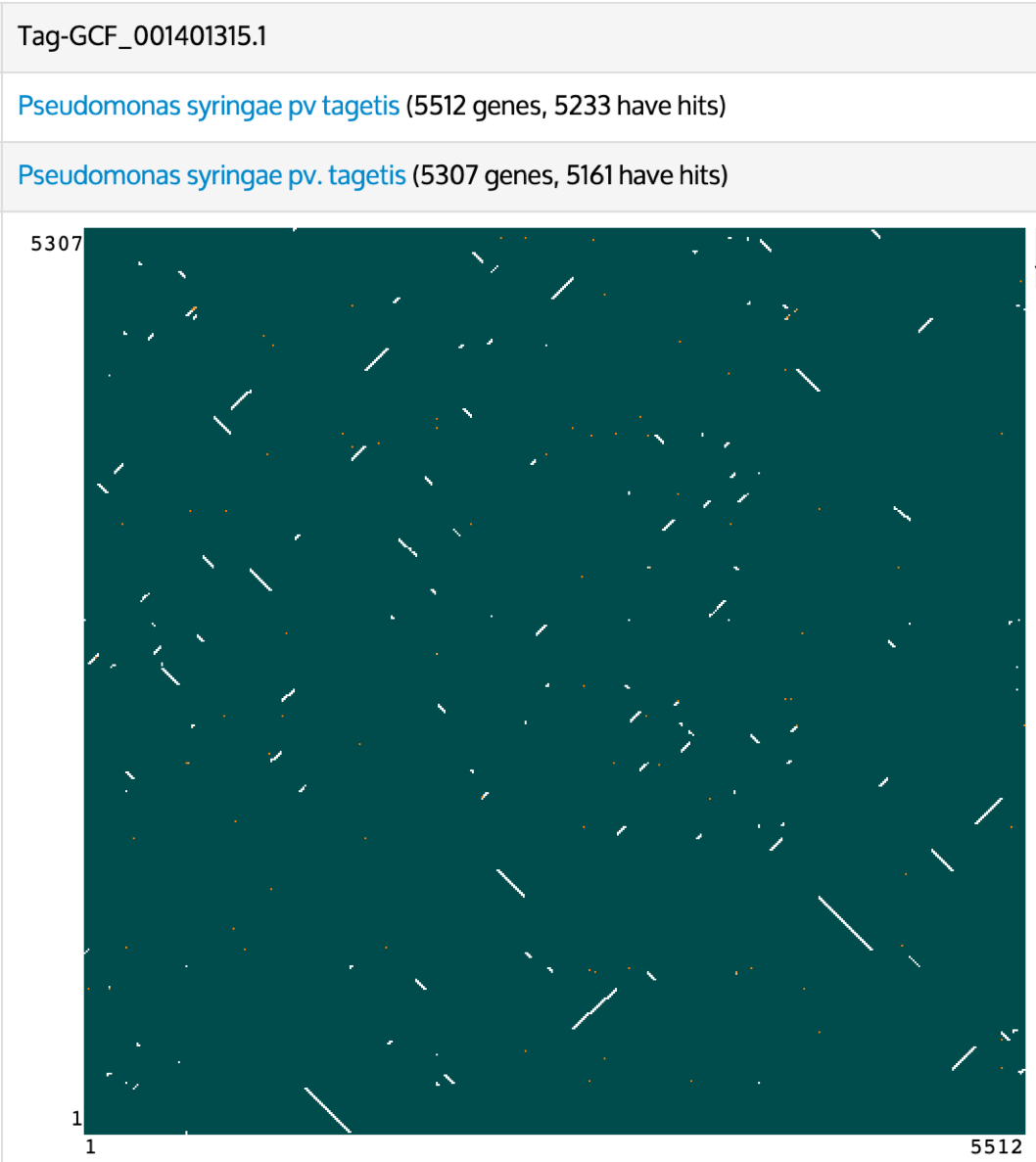


Dot Plot: *P. syringae* pv. *tagetis* EB037 vs. *P. syringae* pv. *tagetis* ICMP 4091

Dot Plot: *P. syringae* pv. *tagetis* EB037 vs. *P. syringae* pv. *syringae* B728A

Dot Plot: *P. syringae* pv. *tagetis* EB037 vs. *P. savastanoi* pv*. phaseolicola* 1448A

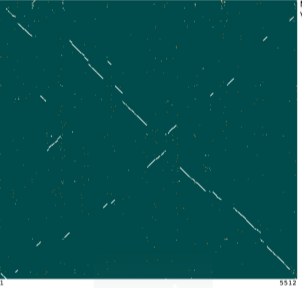


Dot Plot: *P. syringae* pv. *tagetis* EB037 vs. *P. putida* KT2440

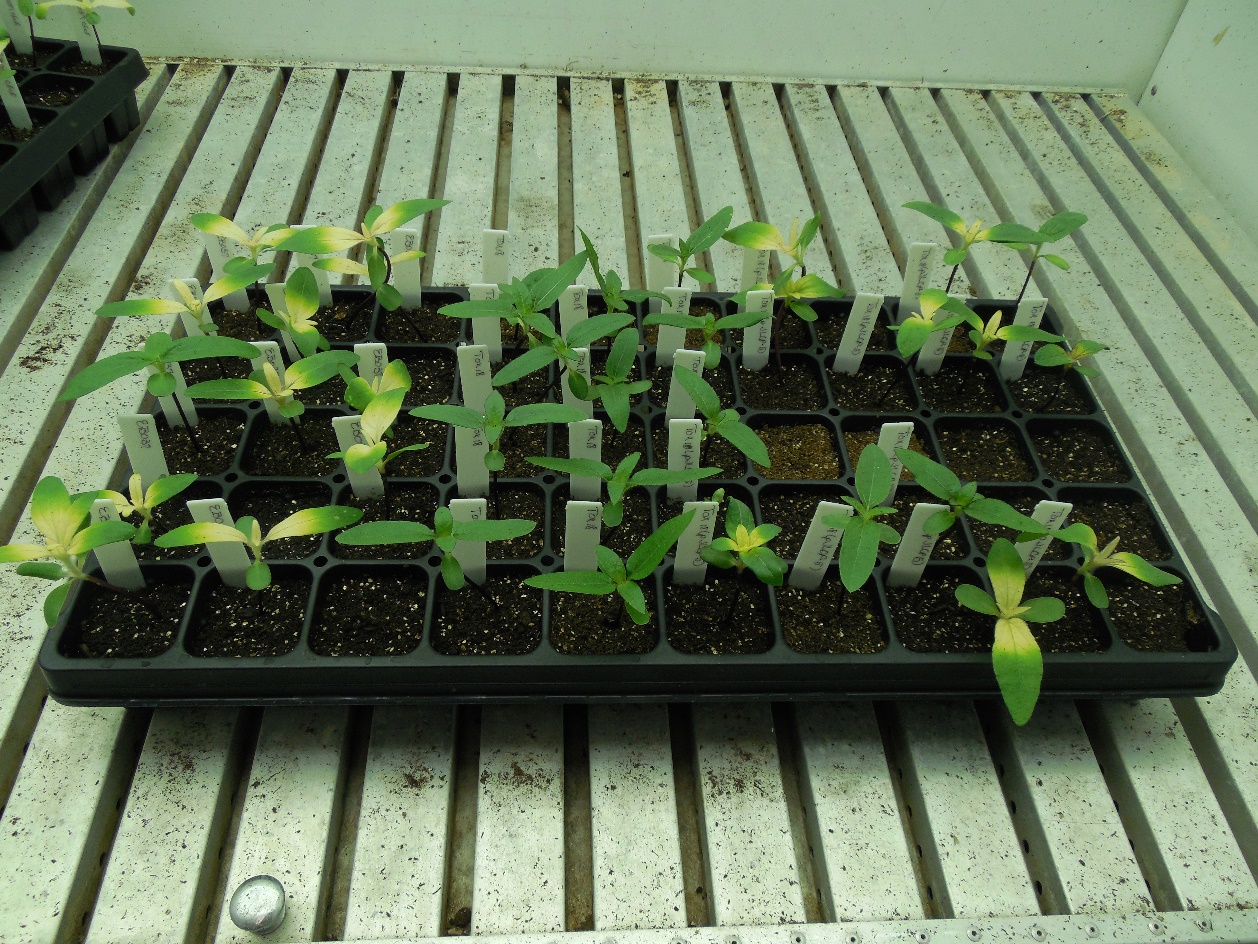


**Supplementary Fig. S2: Image of Sunflower Bioassay**
